# Identification and characterization of gene-based SSR markers in date palm (*Phoenix dactylifera* L.)

**DOI:** 10.1186/1471-2229-12-237

**Published:** 2012-12-15

**Authors:** Yongli Zhao, Roxanne Williams, C S Prakash, Guohao He

**Affiliations:** 1 Department of Agricultural and Environmental Sciences, Tuskegee University, Tuskegee, AL, 36088, USA

**Keywords:** Genic marker, EST sequence, Gene ontology, Polymorphism, Date palm

## Abstract

**Background:**

Date palm (*Phoenix dactylifera* L.) is an important tree in the Middle East and North Africa due to the nutritional value of its fruit. Molecular Breeding would accelerate genetic improvement of fruit tree through marker assisted selection. However, the lack of molecular markers in date palm restricts the application of molecular breeding.

**Results:**

In this study, we analyzed 28,889 EST sequences from the date palm genome database to identify simple-sequence repeats (SSRs) and to develop gene-based markers, i.e. expressed sequence tag-SSRs (EST-SSRs). We identified 4,609 ESTs as containing SSRs, among which, trinucleotide motifs (69.7%) were the most common, followed by tetranucleotide (10.4%) and dinucleotide motifs (9.6%). The motif AG (85.7%) was most abundant in dinucleotides, while motifs AGG (26.8%), AAG (19.3%), and AGC (16.1%) were most common among trinucleotides. A total of 4,967 primer pairs were designed for EST-SSR markers from the computational data. In a follow up laboratory study, we tested a sample of 20 random selected primer pairs for amplification and polymorphism detection using genomic DNA from date palm cultivars. Nearly one-third of these primer pairs detected DNA polymorphism to differentiate the twelve date palm cultivars used. Functional categorization of EST sequences containing SSRs revealed that 3,108 (67.4%) of such ESTs had homology with known proteins.

**Conclusion:**

Date palm EST sequences exhibits a good resource for developing gene-based markers. These genic markers identified in our study may provide a valuable genetic and genomic tool for further genetic research and varietal development in date palm, such as diversity study, QTL mapping, and molecular breeding.

## Background

Date palm (*Phoenix dactylifera* L.) is a dioecious, perennial, monocotyledonous fruit tree belonging to the family Arecaceae, originated in Mesopotamia [1]. Date palm is one of the world’s first cultivated fruit trees representing an ancient group of fruit trees including olive and fig [2]. It is cultivated across the tropical and subtropical areas of South Asia and Africa. As with many other plants, genetic diversity of date palm in its center of origin is threatened by habitat loss due to deforestation, population pressure, and clearance for agriculture development [3]. Moreover, developing elite cultivars using a few genetic materials from gene pool and using offshooting propagation intensively in date palm breeding could further cause the loss of genetic diversity, which resulted in plants vulnerable to genetic erosion. Therefore, preservation and evaluation of genetic diversity are critical and timely concerns in the conservation of date palm germplasm [3].

Genetic variation in the date palm germplasm has been traditionally characterized using morphological descriptors. However such morphological markers are often unreliable and ambigous because of the influence of environmental factors and confounding effects of developmental stage of the plant [4]. Further, detecting genetic variation using morphological traits is time consuming and laborious. The most challenging constraint to tree breeding is the long generation cycle and the many years necessary before the productivity traits are expressed. Thus, any tool that provides a short cut to breeding would be invaluable in the improvement of tree crops such as date palm. For instance, if traits, such as disease and pest resistance, and maleness or females (sex) of the clone, can be determined at an earlier developmental stage using linked molecular markers without the cumbersome phenotyping, it will allow breeders to select elite trees with desirable traits quickly saving time and resource. The advent of molecular biology techniques has provided DNA - based markers for detection of genetic polymorphism in the plant germplasm and also those arise *de novo* due to mutation and somaclonal variation. Many studies report the use of molecular markers to study the genetic diversity and genetic relationship in date palm, including randomly amplified polymorphic DNAs (RAPDs) [5-10], Amplified fragment length polymorphic (AFLP) [11-13], and simple sequence repeat (SSR) [14-17].

The date palm genome has 36 chromosomes (2n=2x=36), and the genome size was estimated between 550 Mb [18] and ~658 Mb long [19]. When compared to many other crop species, there has been relatively little investment in date palm molecular genetics research, resulting in serious constraint of an under-developed infrastructure of genetic and genomic tools. The overall molecular toolbox for data palm is limited although some molecular markers were developed and used including SSR markers, which are not enough to efficiently assess diversity, to construct genetic linkage map, and to use marker-assisted breeding in date palm.

Recent trends in plant research is towards the use of gene-targeted rather than random DNA markers as inexpensive and speedier estimation of genome sequence lately offer enormous potential for the development of such gene-based markers [20]. Gene-based markers are more useful in mapping of quantitative trait loci (QTL), molecular breeding, and gene cloning. The Expressed Sequence Tags (EST)-SSRs are also referred to as genic SSRs. EST-derived SSRs form a valuable genetic marker type, a class of functional markers as a putative function, in mapping candidate genes. Distribution of genic SSRs on the genetic map will show the distribution of genes in the genome. Thus, EST-SSRs have been widely used to construct high-density linkage maps in recent years [21-23] and some EST-SSRs associated with phenotype are useful in marker assisted breeding programs [24,25]. Another important feature of the genic SSR markers is that, unlike genomic SSRs, they are transferable among related species and genera [26].

To date, no gene-based markers have been identified in date palm. The challenge remains not only to identify genes that are responsible for the traits of agronomic interest, but also to identify the gene-related markers that could be used in a breeding program. Therefore, the need is urgent to expand the density and availability of DNA markers, particular gene-based markers for the possibility of molecular breeding in date palm, and to survey the status of molecular characterization of traits of interest across date palm germplasm.

A large number of EST sequences in date palm identified by [19] using *de novo* next-generation sequencing have provided a useful resource to develop gene-based markers. The aim of this study was to characterize genic markers, EST-SSRs, in date palm, to evaluate and compare the frequency and distribution of various types of EST-SSRs in genic sequences, and to develop EST-SSR markers as genetic and genomic tools for date palm.

## Results

### Frequency and distribution of EST-SSRs in date palm

By screening 28,889 assembled date palm EST sequences, we identified 4,609 (16%) as containing SSRs. Because some EST sequences harbored more than one motif, a total of 5,981 various motifs were found from these SSRs (Table 1). Assuming the average length of EST sequences is 500 bp, approximately 14.4 Mb (2.2% of the date palm genome) were analyzed in this study, resulting in a frequency of at least one SSR per 2.4 kb in date palm EST sequences. Among motifs identified, trinucleotide motif (69.7%) was most abundant, followed by tetranucleotide (10.4%) and dinucleotide (9.6%) motifs (Table 1). For dinucleotide motifs, AG (85.7%) was highly abundant, while in trinucleotide motifs, AGG (26.8%) was predominant, followed by AAG (19.3%), and AGC (16.1%) (Table 2).

**Table 1:** Number of SSR motifs mined from EST sequences in Date Palm

**SSR motif**	**Number of SSR**	**Frequency (%)**
Mononucleotide	135	2.3
Dinucleotide	575	9.6
Trinucleotide	4,171	69.7
Tetranucleotide	624	10.4
Pentanucleotide	150	2.5
Hexanucleotide	326	5.5
Total SSRs	5,981	

**Table 2:** Frequency of different type of motif in dinucleotide and trinucleotide SSRs

**Type of motif**	**Number of SSR**	**Frequency (%)**
AT/TA	31	5.4*
AG/GA/CT/TC	493	85.7
AC/CA/TG/GT	47	8.2
GC/CG	4	0.7
AAG/AGA/GAA/CTT/TTC/TCT	805	19.3**
AAT/ATA/TAA/ATT/TTA/TAT	22	0.5
ATG/TGA/GAT/CAT/ATC/TCA	438	10.5
AAC/ACA/CAA/GTT/TTG/TGT	99	2.4
ACC/CCA/CAC/GGT/GTG/TGG	414	9.9
AGG/GGA/GAG/CCT/CTC/TCC	1,119	26.8
AGT/GTA/TAG/ACT/CTA/TAC	38	0.9
AGC/GCA/CAG/GCT/CTG/TGC	673	16.1
ACG/CGA/GAC/CGT/GTC/TCG	178	4.3
GGC/GCG/CGG/GCC/CCG/CGC	385	9.2

*Frequency of motifs in total number of dinucleotide SSRs.

**Frequency of motifs in total number of trinucleotide SSRs.

**Figure 1:**
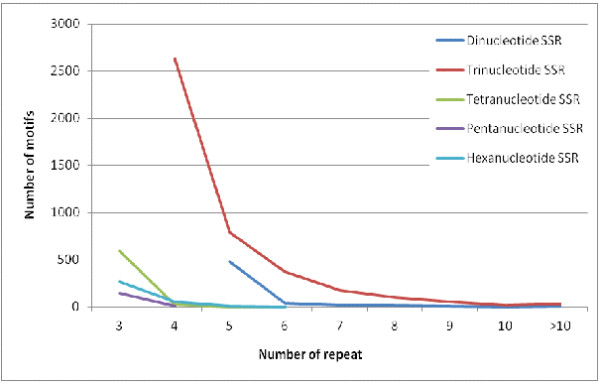
Distribution of EST-SSRs with various motifs across different repeat numbers

To survey the trend of repeat number in various motifs, the distribution of EST-SSRs with various motifs was studied across different repeat numbers. Our results show that the distribution of all di-, tri-, tetra-, penta-, and hexa-nucleotide EST-SSRs was skewed generally to the smaller number of repeats (Figure 1). A few higher repeat numbers were observed in di- and tri-nucleotide SSRs, but in tetra-, penta-, and hexa-nucleotide SSRs, no repeat number was found beyond 6. The average number of repeats in date palm EST-SSR markers was 3.94 repeats per SSR with the range from the low average number (3.03) in pentanucleotide motifs to the high average number (5.26) in dinucleotide motifs.

### EST-SSR marker development and polymorphism detection

We designed 4,967 PCR primer pairs from the chosen 5,981 SSR motifs of date palm; this small reduction was because some EST sequences did not possess enough length of flanking sequences of SSR for designing primers. These EST-SSR primers were named as DPGxxxx, where DPG was referred to date palm gene-based markers and to differentiate from those SSR markers developed from genomic sequences in date palm, such as those developed by [27-29]. The new designed EST-SSR primers were listed in the Additional file 1, along with information on their Tm, GC%, product size, and corresponding EST sequences.

**Figure 2:**
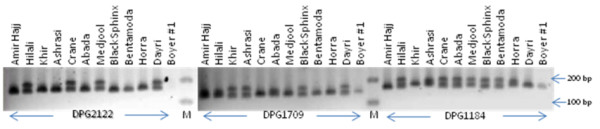
Polymorphism detected by three EST-SSR markers among 12 date palm varltivars

To validate 4,967 EST-SSR markers, we chose a sample of 20 primers randomly from this collection to assess their functionality and ability to detect polymorphism using a template of genomic DNAs isolated from twelve date palm cultivars. Among 20 primer pairs used, one did not produce a visible amplicon, while other 19 generated amplicons of expected product sizes. Six (30%) of 19 markers identified genetic variation among 12 date palm cultivars (Figure 2).

### Functional annotation of the EST sequences containing SSRs

All EST sequences containing SSRs were used in a search of homology for proteins in the NCBI database by the Blast approach. We detected 3,108 sequences (67.4%) as having homology with known proteins, while 787 (17.1%) were homologous to expressed, hypothetical or unknown proteins. The remaining 714 (15.5%) sequences did not possess homology with any known proteins.

**Figure 3:**
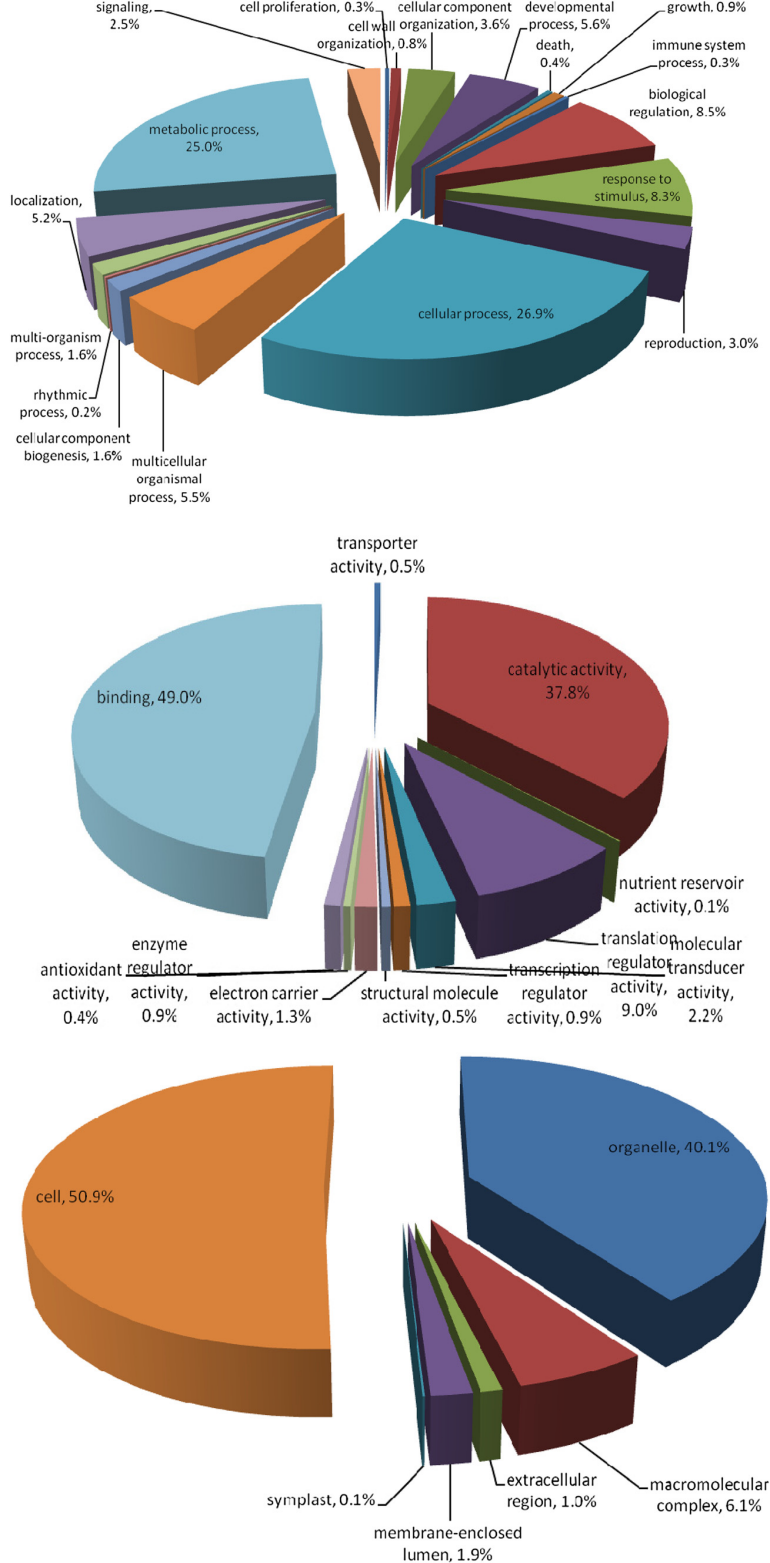
Characterization of date palm EST-SSRs by gene ontology categories

Studies on gene ontology focus on three categories, viz., biological process, molecular function, and cellular component, as representing gene product properties. The gene ontology categorization of date palm EST sequences containing SSRs using Blast2GO in our study revealed that 52% of them classified as involved in biological process for “cellular process” and “metabolic process”. While 49% and 38% of such sequences were homologous to proteins with “binding” and “catalytic activity” of molecular function, respectively. Finally, 51% and 40% of them were homologous to proteins involved with the cellular component, “cell” and “organelle”, respectively (Figure 3).

## Discussion

As the next-generation DNA sequencing is becoming more quicker and inexpensive, vast amounts of sequence data is now being generated exponentially and publicly available, including large number of ESTs from different plant species. These sequences represent a potentially useful resource for mining SSR markers. In this study, we have identified 4,609 date palm EST sequences containing SSRs from a total of 28,889 sequences. The frequency (16%) of SSRs in genic sequences of date palm was lower when compared to other plant species. For instance, the frequency of SSRs detected was 33.3% in citrus [30], 28.4% in castor bean [31], 24% in *Iris*[32]. However, this frequency in data palm (16%) is greater than those detected in oil palm with 6.1% [33]. The SSR density in date palm is one per 2.4 kb, which is also lower than other plant species (one per 1.97 kb in citrus, one per 1.77 kb in castor bean). However, the frequency of SSRs is depended on the criteria used to identify SSRs in the EST sequence database.

The most common dinucleotide SSR motif was AG which comprised of 85.7% dinucleotide motifs in date palm EST sequences. The motif AG is the most abundant and highly polymorphic in both annual and perennial plants including apple and citrus [30,34]. Mun et al. [35] have compared the frequency of motif AG in ESTs vs genomic sequences, and found that the higher frequency of motif AG in EST than in genomic sequences, for *M. truncatular*, soybean, *L. japonicus*, *Arabidopsis*, and rice. Among trinucleotide SSR motifs in date palm, AGG and AAG were the more abundant than other types, while in tetranucleotide SSR motifs, AAAG (19.2%), AAGG (14.3%), and AGGG (13.3%) were more common than other types. Although the role of the SSR motif in the function of plant genes is poorly understood, there is evidence showing that motif AG in the 5’ UTR of the *waxy* gene is related to the amylase content in rice and motif CCG in 5’ UTR in ribosomal protein genes involved in the regulation of fertilization in maize [26]. In date palm, the AG rich content existing genic SSRs and the role of these motifs in the function of genes containing SSRs needs to be further investigated.

Putative functional annotation and categorization of EST sequences containing SSRs in this study revealed that these sequences are involved in various aspects of date palm development. The majorities of transcripts were assigned with “cell” and “organelle” in the cellular component category, involved in “binding” and “catalytic activity” in the molecular function category, and involving in “cellular activity” and “metabolic activity” in the biological process in date palm. Similar results were reported in citrus [30].

Trinucleotide SSRs were the most common, followed by tetra- and dinucleotide SSRs in date palm EST sequences, which is consistent with the most cases in other plant species. The abundance of trinucleotide SSRs in EST sequences was attributed to the tolerance of frameshift mutations in coding regions [26]. There is evidence that EST-SSRs located in coding regions appear to reveal equivalent levels of polymorphism as compared to those located in UTRs [36]. Thus, EST sequences are indeed an excellent resource for mining SSRs in date palm.

While there are a few reports on SSR markers from genomic sequences in date palm, only 56 genomic SSR markers have been identified [27-29]. Increased availability of these markers would aid in the genetic and genomic studies in date palm as they are better tools than RAPD markers because of their co-dominant inheritance, multi-allelic nature, and high reproducibility [5,37-41]. In this study, we report identification of a vast number (4,967) of EST-SSR markers in date palm. Using 20 randomly selected markers, we detected 6 (30%) as identifying polymorphism on a panel of one dozen date palm cultivars. This approach may hold promise for development of a substantial number of informative high-density EST-SSR markers in date palm, large enough to be of value in breeding. These novel markers will not only uplift the repertoire of DNA markers to enrich the genetic and genomic tools, but also facilitate further genomic research in date palm, such as comparative mapping, molecular breeding, and gene cloning because they are derived from transcripts. Such expression profiling can also used to identify agronomically relevant genes based on synteny relationships between plant genomes [20].

Applications and potential uses of EST-SSR markers in plants have proved to be useful in the assessment of genetic diversity [33,42], and also valuable in the identification of gene-inked markers [43,44]. In date palm, lack of gene-related markers has so far limited the application of molecular breeding of this crop. Identification of marker related to gender is especially important in date palm farming as such markers help in easier elimination of male plants. Al-Dous et al. [19] have identified a vast amount of SNPs and a region of the date palm genome linked to gender. The EST-SSRs we reported in this study can potentially be an useful genomic tool in addition to SNPs as they provide a potential resource for association mapping of gender related genes as well as other traits of interest. The large number of gene-based markers can be used in comparative mapping to study colinear order of genes and synteny among close related date palm species due to their high transferability. They can also be utilized to understand genetic diversity in different oases and populations in date palm for its conservation and sustainable use. Once molecular markers linked to desirable traits are identified, marker-assisted selection in breeding will facilitate genetic improvement of this valuable crop species.

## Conclusion

This study sought to ascertain the frequency and distribution of SSRs in the date palm EST sequences and develop gene-based markers EST-SSRs for subsequent application in genetic and genomic studies. The EST-SSR markers identified and characterized in our study may provide an useful tool for research on genetic diversity, gene mapping, and marker-assisted selection in date palm. The functional categorization of date palm EST sequences containing SSR revealed that these ESTs represent many transcribed genes with biology, cellular and molecular function. Therefore, while allowing studies on genetic variation, SSR markers derived from EST sequences also provides information on gene function related to possible phenotypic differences between the date palm cultivars.

## Methods

Publicly available date palm EST sequences were obtained from the database: http://qatar-weill.cornell.edu/research/datepalmGenome/download.html. A total of 28,889 EST sequences were used to mine SSRs with the cutoff of repeat number ≥ 5 for dinucleotide SSRs, ≥ 4 for trinucleotide SSRs, and ≥ 3 for tetra-, penta-, and hexanucleotide SSRs. Primers were designed using BatchPrimer3 [45] with the following conditions: optimum primer length of 20 nucleotides, optimum melting temperature of 50°C, an optimum product size of 150 bp, and an optimum G/C content of 50%. The designed primers were listed in an Additional file 1.

The sequences containing SSR were subjected for functional characterization using Blast2GO [46] to identify the biological process, molecular function, and cellular component ontology for these sequences. The DNA samples from twelve cultivars of date palm used for the polymorphism detection in the lab study were provided by Dr. R. R. Krueger and Dr. M. Keremane of USDA/ARS (Riverside, CA). DNA extraction was performed using the kit of Plant DNA extraction (Qiagen, Valencia, CA) (Table 3).

**Table 3:** Date palm cultivars used for validation of EST-SSR markers

**Accession number**	**Cultivar**	**Country**
PI555400	Abada	USA
PI80781	Amir Hajj	Iraq
PI8739	Ashrasi	Iraq
PI36818	Bentamoda	Sudan
RPHO25	Black Sphinx	USA
PI555420	Boyer#1	USA
PI555421	Crane	USA
PI8567	Dayri	Oman
PI8760	Hilali	Oman
PI15026	Horra	Tunisia
PI11801	Khir	Saudi Arabia
PI74204	Medjool	Morocco
PI10891	Thoory	Algeria

PCR reaction conditions for all newly designed EST-SSR markers were as follows: 25 ng of genomic DNA, 1.5 μM of mixed forward and reverse primers, 1X Buffer (20 mM Tris–HCl, 10 mM (NH_4_)_2_SO_4_, 10 mM KCl, 2 mM MgSO_4_, 0.1% Triton X-100), 0.2 mM dNTPs and 1 U *Taq* polymerase in a total volume of 20 μl. PCR amplification were conducted with the condition: 95°C for 5 min for initial denaturing, followed by 35 cycles of 95°C for 30 sec, 48°C for 30 sec, and 72°C for 1.5 min, and final extension at 72°C for 5 min using Dyad Peltier Thermal Cycler (Bio-Rad Laboratories, Hercules, CA). PCR products were resolved in 2% agarose ultra sieve gel (Shelton Scientific IBI, Peosta, Iowa). Gel staining was performed using EB and visualized by Gel Dec (Bio-Rad Laboratories, Hercules, CA).

## Authors’ contributions

YZ and RW performed data mining analyses and primer design. YZ also screened and developed EST-SSR markers. CSP participated in conception of the study and drafting the manuscript. GH designed and coordinated the study, assisted with statistical genetic analyses, and drafting the manuscript. All authors read and approved the final manuscript.

## Supplementary Material

Additional file 1 List of novel EST-SSR markers along with their primers and motifs. Click here for file
